# Bis[μ-2-(4-hy­droxy­phen­yl)acetato]-κ^3^ *O*,*O*′:*O*;κ^3^ *O*:*O*,*O*′-bis­{aqua­(4,4′-bipyridine-κ*N*)bis­[2-(4-hy­droxy­phen­yl)acetato-κ^2^ *O*,*O*′]thulium(III)} mono­hydrate

**DOI:** 10.1107/S1600536810043898

**Published:** 2010-10-31

**Authors:** Jia-Lu Liu, Jian-Feng Liu, Guo-Liang Zhao

**Affiliations:** a College of Chemistry and Life Sciences, Zhejiang Normal University, Jinhua 321004, People’s Republic of China, and Zhejiang Normal University Xingzhi College, Jinhua 321004, People’s Republic of China

## Abstract

In the title dinuclear complex, [Tm_2_(C_8_H_7_O_3_)_6_(C_10_H_8_N_2_)_2_(H_2_O)_2_]·H_2_O, the Tm^III^ atoms are coordinated by eight O atoms from four 2-(4-hy­droxy­phen­yl)acetate (HPAA) ligands and a water mol­ecule, and one N atom from a 4,4′-bipyridine ligand in a distorted tricapped trigonal–prismatic geometry. While four of the HPAA ligands coordinate to just one Tm atom, the remaining two HPAA ligands bridge the two Tm atoms. In the crystal, O—H⋯O and O—H⋯N hydrogen bonds link the mol­ecules into a three-dimensional network.

## Related literature

For the design, synthesis and applications of carb­oxy­lic metal–organic complexes, see: Liu *et al.* (2010 ▶); Wang & Sevov (2008 ▶); Fang & Zhang (2006 ▶); Wang *et al.* (2010 ▶).
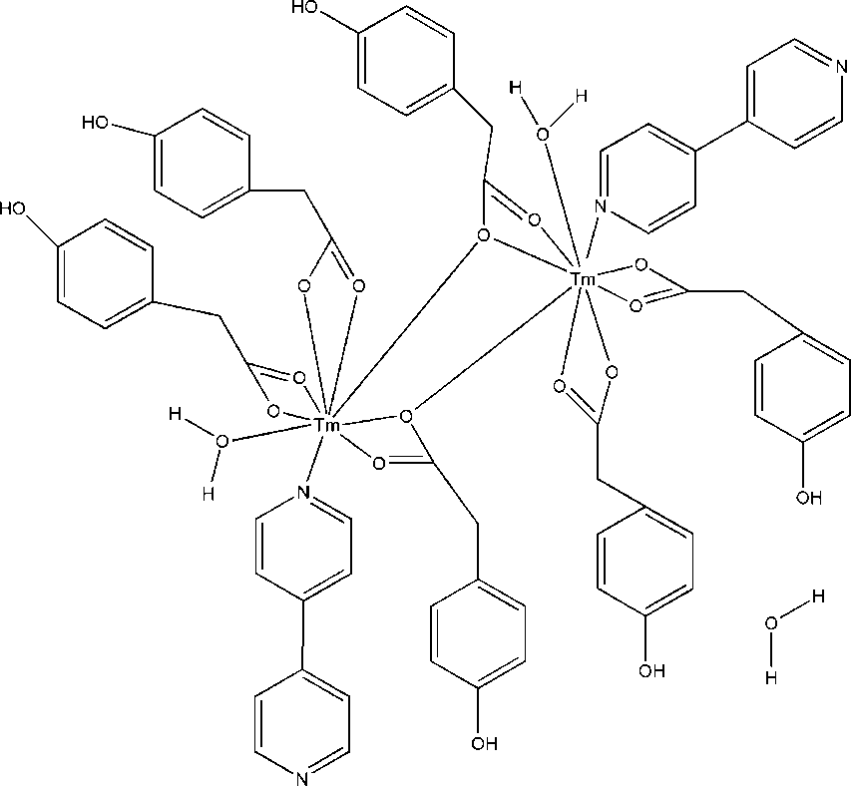


## Experimental

### 

#### Crystal data

[Tm_2_(C_8_H_7_O_3_)_6_(C_10_H_8_N_2_)_2_(H_2_O)_2_]·H_2_O
*M* *_r_* = 1611.10
Triclinic, 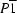

*a* = 11.6999 (1) Å
*b* = 16.1522 (2) Å
*c* = 18.4294 (2) Å
α = 83.294 (1)°
β = 72.374 (1)°
γ = 71.380 (1)°
*V* = 3144.75 (6) Å^3^
*Z* = 2
Mo *K*α radiation
μ = 2.89 mm^−1^
*T* = 296 K
0.22 × 0.06 × 0.05 mm

#### Data collection

Bruker SMART CCD area-detector diffractometer
Absorption correction: multi-scan (*SADABS*; Sheldrick, 1996 ▶) *T*_min_ = 0.824, *T*_max_ = 0.858
48805 measured reflections
14320 independent reflections
11902 reflections with *I* > 2σ(*I*)
*R*_int_ = 0.029

#### Refinement

*R*[*F* ^2^ > 2σ(*F* ^2^)] = 0.023
*wR*(*F* ^2^) = 0.053
*S* = 0.93
14320 reflections
875 parameters
9 restraints
H atoms treated by a mixture of independent and constrained refinement
Δρ_max_ = 0.81 e Å^−3^
Δρ_min_ = −0.72 e Å^−3^

### 

Data collection: *APEX2* (Bruker, 2006 ▶); cell refinement: *SAINT* (Bruker, 2006 ▶); data reduction: *SAINT*; program(s) used to solve structure: *SHELXS97* (Sheldrick, 2008 ▶); program(s) used to refine structure: *SHELXL97* (Sheldrick, 2008 ▶); molecular graphics: *XP* (Sheldrick, 2008 ▶); software used to prepare material for publication: *SHELXL97*.

## Supplementary Material

Crystal structure: contains datablocks I, global. DOI: 10.1107/S1600536810043898/bt5387sup1.cif

Structure factors: contains datablocks I. DOI: 10.1107/S1600536810043898/bt5387Isup2.hkl

Additional supplementary materials:  crystallographic information; 3D view; checkCIF report

## Figures and Tables

**Table 1:** Hydrogen-bond geometry (Å, °)

*D*—H⋯*A*	*D*—H	H⋯*A*	*D*⋯*A*	*D*—H⋯*A*
O3—H3*B*⋯O12^i^	0.82	1.93	2.741 (3)	169
O6—H6*B*⋯O3*W*^ii^	0.82	1.86	2.646 (3)	161
O9—H9*A*⋯O17^iii^	0.82	1.86	2.677 (3)	173
O12—H12*A*⋯O11^iv^	0.82	1.94	2.752 (3)	168
O15—H15*C*⋯O6^v^	0.82	1.90	2.717 (3)	175
O18—H18*B*⋯O9^ii^	0.82	1.95	2.766 (3)	173
O2*W*—H2*WA*⋯O5	0.83 (4)	1.99 (2)	2.733 (3)	150 (4)
O2*W*—H2*WB*⋯N2^ii^	0.82 (2)	2.04 (2)	2.836 (3)	164 (4)
O3*W*—H3*WB*⋯O3	0.84 (4)	1.97 (4)	2.793 (3)	166 (4)
O1*W*—H1*WA*⋯O13	0.84 (5)	1.95 (2)	2.733 (2)	157 (4)
O1*W*—H1*WB*⋯N4^i^	0.83 (2)	1.96 (2)	2.780 (3)	169 (4)
O3*W*—H3*WA*⋯O1^vi^	0.85 (4)	1.93 (4)	2.778 (3)	174 (5)

Symmetry codes: (i) 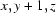; (ii) 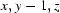; (iii) 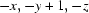; (iv) 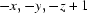; (v) 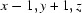; (vi) 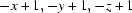.

**Fig. 1.**
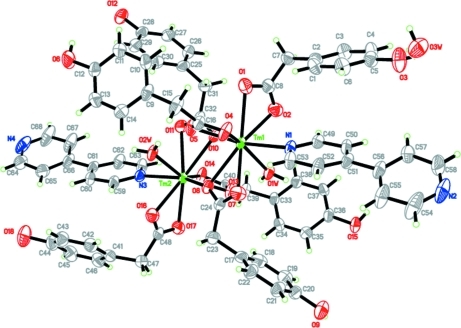
The molecular structure of the title complex, showing the atom-labelling scheme. Displacement ellipsoids are drawn at the 50% probability level.

## supplementary crystallographic information

### Comment

The design and synthesis of carboxylic metal-organic complexes have been of increasing interest for decades owing to their potential practical applications in fluorescence and magnetism (Wang  *et al.*, 2010; Fang  & Zhang, 2006; Wang  & Sevov, 2008; Liu  *et al.*, 2010). The title compound consists of six  PAA  ligands, two 4,4'-bipyridine(bipy) molecules and three water molecules. In the binuclear complex, each Tm atom is coordinated by seven O atoms from four PAA ligands, one N atom from a 4,4'-bipyridine ligand and one O atom from a water molecule. The Tm atoms are nine coordinated. Four PAA ligands coordinate to one Tm centre and two of them bridge two Tm centres (Fig. 1). The thulium^III^ atom is in a distorted capped pentagonal prism environment. The Tm–O bond lengths range from 2.3052 (16) Å-2.5406 (18) Å. The Tm–N distances range from 2.507 (2) Å-2.525 (2) Å. The Tm–O(water) bond lengths range from 2.3429 (18) Å-2.3454 (18) Å, which is slightly shorter than the other Tm–O bonds. In addition, there are plenty of hydrogen bonds in the crystal structure. The occurrence of numerous O–H···O involving coordinated and non-coordinated water molecules build up an intricated three dimensionnal network.

### Experimental

All reagents and solvents used were of commercially available quality and were not purified. *p*-hydroxyphenylacetic acid (HPAA) (0.456 g, 3 mmol) and sodium hydroxide (0.12 g, 3 mmol) were mixed together in water (10 ml), then Tm[(NO_3_)_3_] (0.330 g, 1 mmol) dissolved in water (10 ml) was added into the above solution. After stirring for one hour, an ethanol (5 ml) solution of 4,4'-bipyridine (0.156 g, 1 mmol) was slowly dripped into the above solution with stirring for three hours. After filtration, the filtrate was allowed to stand at room temperature, and single crystals were obtained after a week.

### Refinement

H atoms excluding the water H atoms were geometrically positioned and treated as riding with C–H = 0.97 Å (methylene) or 0.93 Å (aromatic) and O–H = 0.82 Å with *U*_iso_(H) = 1.2*U*_eq_(C) or *U*_iso_(H) = 1.5*U*_eq_(O). The H atoms of the water molecules were located in a difference Fourier map. Their coordinates were refined using restraints [O–H = 0.82 (1)Å and H···H = 1.39 (2) Å] with *U*_iso_(H) = 1.5*U*_eq_(O).

### Crystal data

**Table:**

[Tm_2_(C_8_H_7_O_3_)_6_(C_10_H_8_N_2_)_2_(H_2_O)_2_]·H_2_O	*Z* = 2
*M**_r_* = 1611.10	*F*(000) = 1612
Triclinic, *P*1	*D*_x_ = 1.701 Mg m^−^^3^
Hall symbol: -P 1	Mo *K*α radiation, λ = 0.71073 Å
*a* = 11.6999 (1) Å	Cell parameters from 9775 reflections
*b* = 16.1522 (2) Å	θ = 1.7–27.7°
*c* = 18.4294 (2) Å	µ = 2.89 mm^−^^1^
α = 83.294 (1)°	*T* = 296 K
β = 72.374 (1)°	Block, colourless
γ = 71.380 (1)°	0.22 × 0.06 × 0.05 mm
*V* = 3144.75 (6) Å^3^	

### Data collection

**Table:**

Bruker SMART CCD area-detector diffractometer	14320 independent reflections
Radiation source: fine-focus sealed tube	11902 reflections with *I* > 2σ(*I*)
graphite	*R*_int_ = 0.029
phi and ω scans	θ_max_ = 27.6°, θ_min_ = 1.2°
Absorption correction: empirical (using intensity measurements) (*SADABS*; Sheldrick, 1996)	*h* = −15→15
*T*_min_ = 0.824, *T*_max_ = 0.858	*k* = −21→21
48805 measured reflections	*l* = −24→23

### Refinement

**Table:**

Refinement on *F*^2^	Primary atom site location: structure-invariant direct methods
Least-squares matrix: full	Secondary atom site location: difference Fourier map
*R*[*F*^2^ > 2σ(*F*^2^)] = 0.023	Hydrogen site location: inferred from neighbouring sites
*wR*(*F*^2^) = 0.053	H atoms treated by a mixture of independent and constrained refinement
*S* = 0.93	*w* = 1/[σ^2^(*F*_o_^2^) + (0.0219*P*)^2^ + 2.5538*P*] where *P* = (*F*_o_^2^ + 2*F*_c_^2^)/3
14320 reflections	(Δ/σ)_max_ = 0.002
875 parameters	Δρ_max_ = 0.81 e Å^−^^3^
9 restraints	Δρ_min_ = −0.72 e Å^−^^3^

### Special details

**Table:**

Geometry. All e.s.d.'s (except the e.s.d. in the dihedral angle between two l.s. planes) are estimated using the full covariance matrix. The cell e.s.d.'s are taken into account individually in the estimation of e.s.d.'s in distances, angles and torsion angles; correlations between e.s.d.'s in cell parameters are only used when they are defined by crystal symmetry. An approximate (isotropic) treatment of cell e.s.d.'s is used for estimating e.s.d.'s involving l.s. planes.
Refinement. Refinement of *F*^2^ against ALL reflections. The weighted *R*-factor *wR* and goodness of fit *S* are based on *F*^2^, conventional *R*-factors *R* are based on *F*, with *F* set to zero for negative *F*^2^. The threshold expression of *F*^2^ > σ(*F*^2^) is used only for calculating *R*-factors(gt) *etc*. and is not relevant to the choice of reflections for refinement. *R*-factors based on *F*^2^ are statistically about twice as large as those based on *F*, and *R*- factors based on ALL data will be even larger.

### Fractional atomic coordinates and isotropic or equivalent isotropic displacement parameters (Å^2^)

**Table:**

	*x*	*y*	*z*	*U*_iso_*/*U*_eq_	
Tm1	0.272893 (10)	0.363596 (6)	0.281576 (6)	0.02507 (3)	
Tm2	0.131092 (10)	0.207933 (6)	0.198069 (6)	0.02443 (3)	
N1	0.3116 (2)	0.50754 (13)	0.28166 (13)	0.0322 (5)	
N2	0.3909 (3)	0.92956 (18)	0.2257 (2)	0.0645 (9)	
N3	0.0886 (2)	0.06565 (13)	0.19142 (13)	0.0316 (5)	
N4	0.0372 (3)	−0.36400 (18)	0.2271 (2)	0.0660 (10)	
C1	0.2254 (3)	0.50686 (18)	0.51505 (15)	0.0344 (6)	
C2	0.3414 (3)	0.5135 (2)	0.47165 (17)	0.0431 (7)	
H2A	0.3982	0.4666	0.4423	0.052*	
C3	0.3746 (3)	0.5887 (2)	0.47106 (18)	0.0469 (8)	
H3A	0.4527	0.5923	0.4409	0.056*	
C4	0.2926 (3)	0.65763 (19)	0.51483 (18)	0.0415 (7)	
C5	0.1780 (3)	0.6518 (2)	0.55957 (19)	0.0469 (8)	
H5A	0.1228	0.6981	0.5902	0.056*	
C6	0.1444 (3)	0.57766 (19)	0.55934 (17)	0.0424 (7)	
H6A	0.0659	0.5749	0.5894	0.051*	
C7	0.1871 (3)	0.42530 (19)	0.51734 (16)	0.0400 (7)	
H7A	0.2314	0.3800	0.5470	0.048*	
H7B	0.0977	0.4379	0.5422	0.048*	
C8	0.2159 (3)	0.39289 (19)	0.43848 (16)	0.0374 (7)	
C9	0.6223 (3)	0.09082 (17)	0.30142 (18)	0.0381 (7)	
C10	0.6025 (3)	0.05960 (19)	0.37533 (19)	0.0499 (8)	
H10A	0.5885	0.0971	0.4139	0.060*	
C11	0.6029 (3)	−0.0259 (2)	0.39428 (18)	0.0507 (8)	
H11A	0.5899	−0.0456	0.4447	0.061*	
C12	0.6226 (3)	−0.08143 (17)	0.33756 (17)	0.0382 (7)	
C13	0.6405 (3)	−0.05130 (19)	0.26372 (18)	0.0470 (8)	
H13A	0.6528	−0.0886	0.2254	0.056*	
C14	0.6403 (3)	0.03341 (19)	0.24605 (18)	0.0453 (7)	
H14A	0.6525	0.0528	0.1956	0.054*	
C15	0.6242 (3)	0.18338 (18)	0.2810 (2)	0.0490 (8)	
H15A	0.6894	0.1832	0.2336	0.059*	
H15B	0.6471	0.2045	0.3201	0.059*	
C16	0.5023 (3)	0.24669 (17)	0.27218 (16)	0.0330 (6)	
C17	0.2733 (3)	0.45381 (19)	0.01336 (16)	0.0412 (7)	
C18	0.1479 (3)	0.5009 (2)	0.02823 (17)	0.0473 (8)	
H18A	0.0874	0.4741	0.0550	0.057*	
C19	0.1101 (3)	0.5874 (2)	0.00401 (17)	0.0443 (7)	
H19A	0.0250	0.6180	0.0144	0.053*	
C20	0.1992 (3)	0.62794 (18)	−0.03564 (16)	0.0372 (6)	
C21	0.3254 (3)	0.58146 (19)	−0.05033 (17)	0.0411 (7)	
H21A	0.3860	0.6084	−0.0766	0.049*	
C22	0.3618 (3)	0.49529 (18)	−0.02619 (17)	0.0412 (7)	
H22A	0.4469	0.4646	−0.0366	0.049*	
C23	0.3133 (4)	0.3601 (2)	0.04063 (17)	0.0562 (10)	
H23A	0.2593	0.3297	0.0320	0.067*	
H23B	0.3987	0.3317	0.0110	0.067*	
C24	0.3075 (3)	0.35233 (16)	0.12355 (15)	0.0314 (6)	
C25	0.1425 (3)	0.11458 (18)	0.47590 (16)	0.0367 (7)	
C26	0.0402 (3)	0.10736 (19)	0.53550 (17)	0.0424 (7)	
H26A	−0.0283	0.1566	0.5510	0.051*	
C27	0.0384 (3)	0.02770 (19)	0.57234 (16)	0.0405 (7)	
H27A	−0.0307	0.0238	0.6125	0.049*	
C28	0.1387 (3)	−0.04523 (18)	0.54933 (16)	0.0348 (6)	
C29	0.2426 (3)	−0.03927 (19)	0.49047 (17)	0.0410 (7)	
H29A	0.3111	−0.0886	0.4751	0.049*	
C30	0.2432 (3)	0.04091 (19)	0.45486 (17)	0.0412 (7)	
H30A	0.3134	0.0451	0.4157	0.049*	
C31	0.1453 (3)	0.19992 (18)	0.43314 (16)	0.0422 (7)	
H31A	0.0820	0.2472	0.4640	0.051*	
H31B	0.2266	0.2080	0.4266	0.051*	
C32	0.1228 (2)	0.20683 (16)	0.35645 (15)	0.0291 (6)	
C33	−0.2525 (2)	0.47879 (16)	0.24482 (16)	0.0306 (6)	
C34	−0.2101 (3)	0.51760 (18)	0.17474 (16)	0.0382 (7)	
H34A	−0.1722	0.4833	0.1317	0.046*	
C35	−0.2230 (3)	0.60530 (18)	0.16748 (17)	0.0403 (7)	
H35A	−0.1942	0.6297	0.1198	0.048*	
C36	−0.2785 (3)	0.65763 (17)	0.23061 (17)	0.0374 (6)	
C37	−0.3210 (3)	0.62041 (18)	0.30082 (16)	0.0396 (7)	
H37A	−0.3586	0.6548	0.3438	0.048*	
C38	−0.3077 (3)	0.53177 (17)	0.30730 (16)	0.0370 (6)	
H38A	−0.3367	0.5074	0.3549	0.044*	
C39	−0.2391 (2)	0.38273 (16)	0.25170 (17)	0.0358 (6)	
H39A	−0.2838	0.3699	0.3032	0.043*	
H39B	−0.2799	0.3697	0.2176	0.043*	
C40	−0.1067 (2)	0.32280 (16)	0.23423 (15)	0.0284 (5)	
C41	0.2331 (3)	0.05730 (18)	−0.04364 (15)	0.0384 (7)	
C42	0.3523 (3)	0.00665 (19)	−0.04008 (16)	0.0407 (7)	
H42A	0.4084	0.0340	−0.0351	0.049*	
C43	0.3894 (3)	−0.0831 (2)	−0.04371 (17)	0.0451 (7)	
H43A	0.4703	−0.1155	−0.0424	0.054*	
C44	0.3065 (3)	−0.1248 (2)	−0.04932 (18)	0.0459 (7)	
C45	0.1883 (3)	−0.0759 (2)	−0.05398 (18)	0.0492 (8)	
H45A	0.1325	−0.1036	−0.0588	0.059*	
C46	0.1527 (3)	0.0138 (2)	−0.05150 (17)	0.0449 (7)	
H46A	0.0730	0.0460	−0.0552	0.054*	
C47	0.1897 (3)	0.15558 (19)	−0.03641 (17)	0.0455 (7)	
H47A	0.2525	0.1796	−0.0712	0.055*	
H47B	0.1123	0.1797	−0.0510	0.055*	
C48	0.1678 (3)	0.18238 (17)	0.04343 (16)	0.0355 (6)	
C49	0.2289 (3)	0.57610 (17)	0.32173 (16)	0.0349 (6)	
H49A	0.1558	0.5686	0.3559	0.042*	
C50	0.2461 (3)	0.65735 (17)	0.31520 (17)	0.0372 (6)	
H50A	0.1856	0.7030	0.3446	0.045*	
C51	0.3534 (3)	0.67103 (17)	0.26491 (17)	0.0354 (6)	
C52	0.4413 (3)	0.59957 (18)	0.22479 (18)	0.0424 (7)	
H52A	0.5163	0.6051	0.1915	0.051*	
C53	0.4172 (3)	0.52025 (18)	0.23434 (18)	0.0394 (7)	
H53A	0.4772	0.4732	0.2066	0.047*	
C54	0.4488 (4)	0.8688 (2)	0.1743 (3)	0.0765 (13)	
H54A	0.4979	0.8839	0.1278	0.092*	
C55	0.4421 (3)	0.7834 (2)	0.1839 (2)	0.0607 (10)	
H55A	0.4841	0.7436	0.1448	0.073*	
C56	0.3716 (3)	0.75945 (17)	0.25284 (19)	0.0408 (7)	
C57	0.3143 (4)	0.8218 (2)	0.3081 (2)	0.0612 (10)	
H57A	0.2681	0.8083	0.3561	0.073*	
C58	0.3261 (4)	0.9047 (2)	0.2914 (2)	0.0694 (11)	
H58A	0.2853	0.9460	0.3293	0.083*	
C59	0.1759 (3)	−0.00021 (17)	0.14963 (16)	0.0369 (6)	
H59A	0.2497	0.0096	0.1185	0.044*	
C60	0.1618 (3)	−0.08216 (17)	0.15046 (17)	0.0417 (7)	
H60A	0.2245	−0.1253	0.1194	0.050*	
C61	0.0551 (3)	−0.10025 (17)	0.19724 (16)	0.0360 (6)	
C62	−0.0371 (3)	−0.03130 (18)	0.23823 (18)	0.0419 (7)	
H62A	−0.1123	−0.0392	0.2690	0.050*	
C63	−0.0176 (3)	0.04962 (18)	0.23345 (17)	0.0390 (7)	
H63A	−0.0818	0.0952	0.2609	0.047*	
C64	0.1173 (5)	−0.3399 (2)	0.1670 (2)	0.0687 (12)	
H64A	0.1722	−0.3822	0.1322	0.082*	
C65	0.1234 (4)	−0.2547 (2)	0.1534 (2)	0.0609 (10)	
H65A	0.1800	−0.2408	0.1099	0.073*	
C66	0.0447 (3)	−0.19095 (17)	0.20522 (19)	0.0428 (7)	
C67	−0.0402 (3)	−0.2154 (2)	0.2672 (2)	0.0607 (10)	
H67A	−0.0962	−0.1745	0.3030	0.073*	
C68	−0.0408 (4)	−0.3015 (2)	0.2753 (3)	0.0711 (12)	
H68A	−0.0994	−0.3166	0.3170	0.085*	
O1W	0.09088 (18)	0.45657 (11)	0.25384 (12)	0.0359 (4)	
O1	0.3149 (2)	0.33406 (14)	0.41036 (12)	0.0498 (5)	
O2W	0.32075 (18)	0.11468 (11)	0.21618 (13)	0.0378 (5)	
O2	0.1408 (2)	0.42768 (13)	0.39911 (11)	0.0422 (5)	
O3	0.3283 (2)	0.73172 (15)	0.51250 (15)	0.0641 (7)	
H3B	0.2698	0.7695	0.5384	0.076 (14)*	
O3W	0.5549 (3)	0.76744 (16)	0.49078 (15)	0.0607 (6)	
O4	0.49211 (18)	0.32622 (12)	0.26334 (13)	0.0439 (5)	
O5	0.41074 (17)	0.21987 (11)	0.27557 (12)	0.0370 (4)	
O6	0.6261 (2)	−0.16739 (13)	0.35175 (13)	0.0607 (7)	
H6B	0.5991	−0.1756	0.3977	0.091*	
O7	0.3518 (2)	0.39828 (13)	0.15010 (11)	0.0449 (5)	
O8	0.25547 (16)	0.29964 (11)	0.16721 (10)	0.0310 (4)	
O9	0.1681 (2)	0.71282 (13)	−0.06079 (13)	0.0542 (6)	
H9A	0.0972	0.7271	−0.0661	0.081*	
O10	0.14550 (16)	0.26964 (11)	0.31179 (10)	0.0293 (4)	
O11	0.08385 (19)	0.15314 (12)	0.33458 (11)	0.0370 (4)	
O12	0.1411 (2)	−0.12635 (12)	0.58313 (12)	0.0458 (5)	
H12A	0.0692	−0.1267	0.6059	0.069*	
O13	−0.01256 (17)	0.35083 (11)	0.20618 (12)	0.0365 (4)	
O14	−0.08821 (18)	0.24205 (11)	0.24648 (12)	0.0402 (5)	
O15	−0.2883 (2)	0.74424 (12)	0.21972 (13)	0.0541 (6)	
H15C	−0.3147	0.7679	0.2611	0.081*	
O16	0.25669 (19)	0.15830 (12)	0.07359 (11)	0.0389 (5)	
O17	0.0615 (2)	0.22751 (13)	0.08142 (12)	0.0473 (5)	
O18	0.3466 (3)	−0.21394 (14)	−0.05109 (16)	0.0708 (7)	
H18B	0.2902	−0.2316	−0.0546	0.106*	
H2WA	0.371 (3)	0.130 (2)	0.231 (2)	0.106*	
H3WB	0.494 (3)	0.748 (3)	0.498 (3)	0.106*	
H1WA	0.043 (4)	0.437 (2)	0.240 (3)	0.106*	
H1WB	0.065 (4)	0.5101 (12)	0.246 (3)	0.106*	
H3WA	0.596 (3)	0.733 (3)	0.519 (2)	0.106*	
H2WB	0.353 (4)	0.0615 (12)	0.211 (3)	0.106*	

### Atomic displacement parameters (Å^2^)

**Table:**

	*U*^11^	*U*^22^	*U*^33^	*U*^12^	*U*^13^	*U*^23^
Tm1	0.02738 (6)	0.01615 (5)	0.03496 (7)	−0.00853 (4)	−0.01254 (5)	0.00258 (4)
Tm2	0.02646 (6)	0.01500 (5)	0.03413 (7)	−0.00686 (4)	−0.01144 (5)	0.00029 (4)
N1	0.0337 (12)	0.0206 (11)	0.0457 (14)	−0.0097 (9)	−0.0153 (11)	0.0017 (9)
N2	0.0582 (19)	0.0284 (15)	0.119 (3)	−0.0187 (14)	−0.042 (2)	0.0129 (17)
N3	0.0385 (13)	0.0215 (11)	0.0381 (13)	−0.0103 (10)	−0.0154 (10)	0.0022 (9)
N4	0.087 (2)	0.0300 (15)	0.114 (3)	−0.0287 (16)	−0.072 (2)	0.0198 (17)
C1	0.0391 (16)	0.0379 (15)	0.0296 (15)	−0.0138 (13)	−0.0118 (12)	−0.0016 (11)
C2	0.0403 (17)	0.0452 (18)	0.0412 (17)	−0.0114 (14)	−0.0050 (14)	−0.0132 (13)
C3	0.0362 (16)	0.0513 (19)	0.054 (2)	−0.0176 (15)	−0.0059 (14)	−0.0078 (15)
C4	0.0399 (17)	0.0378 (16)	0.0517 (19)	−0.0138 (14)	−0.0168 (14)	−0.0029 (13)
C5	0.0407 (17)	0.0363 (16)	0.057 (2)	−0.0048 (14)	−0.0066 (15)	−0.0145 (14)
C6	0.0337 (16)	0.0455 (17)	0.0459 (18)	−0.0127 (14)	−0.0050 (13)	−0.0070 (14)
C7	0.0514 (18)	0.0393 (16)	0.0315 (15)	−0.0187 (14)	−0.0094 (13)	−0.0006 (12)
C8	0.0460 (17)	0.0338 (15)	0.0385 (16)	−0.0243 (14)	−0.0084 (14)	0.0011 (12)
C9	0.0277 (14)	0.0258 (14)	0.064 (2)	−0.0035 (11)	−0.0231 (14)	0.0018 (13)
C10	0.071 (2)	0.0279 (15)	0.054 (2)	−0.0043 (15)	−0.0293 (18)	−0.0114 (14)
C11	0.071 (2)	0.0350 (17)	0.0412 (18)	−0.0078 (16)	−0.0178 (17)	−0.0022 (13)
C12	0.0441 (17)	0.0265 (14)	0.0440 (17)	−0.0096 (13)	−0.0132 (14)	−0.0016 (12)
C13	0.065 (2)	0.0352 (16)	0.0412 (18)	−0.0131 (15)	−0.0142 (16)	−0.0080 (13)
C14	0.057 (2)	0.0365 (16)	0.0399 (17)	−0.0118 (15)	−0.0148 (15)	0.0037 (13)
C15	0.0364 (16)	0.0281 (15)	0.090 (3)	−0.0102 (13)	−0.0308 (17)	0.0073 (15)
C16	0.0335 (15)	0.0265 (14)	0.0404 (16)	−0.0085 (12)	−0.0142 (12)	0.0021 (11)
C17	0.069 (2)	0.0368 (16)	0.0292 (15)	−0.0276 (16)	−0.0193 (15)	0.0036 (12)
C18	0.062 (2)	0.059 (2)	0.0337 (17)	−0.0393 (18)	−0.0098 (15)	−0.0007 (14)
C19	0.0399 (17)	0.0519 (19)	0.0445 (18)	−0.0154 (15)	−0.0131 (14)	−0.0061 (14)
C20	0.0484 (18)	0.0317 (15)	0.0390 (16)	−0.0148 (13)	−0.0207 (14)	0.0006 (12)
C21	0.0465 (18)	0.0364 (16)	0.0471 (18)	−0.0213 (14)	−0.0158 (14)	0.0063 (13)
C22	0.0504 (18)	0.0337 (15)	0.0472 (18)	−0.0170 (14)	−0.0225 (15)	0.0048 (13)
C23	0.106 (3)	0.0359 (17)	0.0385 (18)	−0.0356 (19)	−0.0250 (19)	0.0053 (13)
C24	0.0382 (15)	0.0238 (13)	0.0334 (15)	−0.0113 (12)	−0.0113 (12)	0.0028 (11)
C25	0.0550 (18)	0.0313 (15)	0.0361 (16)	−0.0218 (14)	−0.0247 (14)	0.0092 (12)
C26	0.0519 (19)	0.0309 (15)	0.0444 (18)	−0.0107 (14)	−0.0162 (15)	0.0022 (12)
C27	0.0453 (17)	0.0399 (16)	0.0355 (16)	−0.0164 (14)	−0.0092 (13)	0.0061 (13)
C28	0.0448 (17)	0.0314 (14)	0.0349 (15)	−0.0169 (13)	−0.0175 (13)	0.0063 (11)
C29	0.0410 (17)	0.0380 (16)	0.0421 (17)	−0.0108 (13)	−0.0117 (14)	0.0044 (13)
C30	0.0460 (18)	0.0430 (17)	0.0391 (17)	−0.0229 (15)	−0.0129 (14)	0.0111 (13)
C31	0.064 (2)	0.0348 (16)	0.0419 (17)	−0.0265 (15)	−0.0256 (15)	0.0075 (13)
C32	0.0303 (14)	0.0212 (12)	0.0367 (15)	−0.0102 (11)	−0.0096 (11)	0.0039 (10)
C33	0.0268 (13)	0.0241 (13)	0.0411 (16)	−0.0035 (11)	−0.0128 (12)	−0.0044 (11)
C34	0.0420 (17)	0.0319 (15)	0.0365 (16)	−0.0052 (13)	−0.0077 (13)	−0.0104 (12)
C35	0.0502 (18)	0.0350 (16)	0.0363 (16)	−0.0146 (14)	−0.0122 (14)	0.0024 (12)
C36	0.0421 (17)	0.0239 (13)	0.0491 (18)	−0.0073 (12)	−0.0188 (14)	−0.0039 (12)
C37	0.0460 (17)	0.0325 (15)	0.0376 (16)	−0.0040 (13)	−0.0127 (14)	−0.0109 (12)
C38	0.0383 (16)	0.0306 (15)	0.0373 (16)	−0.0050 (12)	−0.0095 (13)	−0.0003 (12)
C39	0.0295 (14)	0.0245 (13)	0.0526 (18)	−0.0059 (11)	−0.0125 (13)	−0.0018 (12)
C40	0.0305 (14)	0.0233 (13)	0.0342 (15)	−0.0064 (11)	−0.0142 (11)	−0.0032 (10)
C41	0.0494 (18)	0.0352 (15)	0.0292 (15)	−0.0127 (14)	−0.0083 (13)	−0.0032 (12)
C42	0.0472 (18)	0.0412 (17)	0.0393 (17)	−0.0210 (14)	−0.0114 (14)	−0.0020 (13)
C43	0.0479 (18)	0.0408 (17)	0.0487 (19)	−0.0148 (15)	−0.0148 (15)	−0.0014 (14)
C44	0.055 (2)	0.0364 (17)	0.0451 (18)	−0.0163 (15)	−0.0087 (15)	−0.0047 (13)
C45	0.051 (2)	0.0486 (19)	0.054 (2)	−0.0241 (16)	−0.0103 (16)	−0.0090 (15)
C46	0.0425 (17)	0.0482 (19)	0.0449 (18)	−0.0119 (15)	−0.0128 (14)	−0.0088 (14)
C47	0.064 (2)	0.0355 (16)	0.0349 (17)	−0.0086 (15)	−0.0172 (15)	−0.0005 (12)
C48	0.0492 (18)	0.0203 (13)	0.0374 (16)	−0.0139 (13)	−0.0110 (14)	0.0043 (11)
C49	0.0378 (15)	0.0284 (14)	0.0399 (16)	−0.0125 (12)	−0.0111 (13)	0.0010 (11)
C50	0.0416 (16)	0.0224 (13)	0.0491 (18)	−0.0081 (12)	−0.0156 (14)	−0.0032 (12)
C51	0.0382 (15)	0.0238 (13)	0.0538 (18)	−0.0127 (12)	−0.0249 (14)	0.0044 (12)
C52	0.0346 (16)	0.0271 (14)	0.065 (2)	−0.0129 (12)	−0.0104 (14)	0.0025 (13)
C53	0.0334 (15)	0.0248 (14)	0.0591 (19)	−0.0094 (12)	−0.0094 (14)	−0.0048 (13)
C54	0.049 (2)	0.042 (2)	0.125 (4)	−0.0209 (18)	−0.007 (2)	0.022 (2)
C55	0.0457 (19)	0.0301 (17)	0.093 (3)	−0.0131 (15)	−0.0008 (19)	0.0034 (17)
C56	0.0402 (16)	0.0247 (14)	0.066 (2)	−0.0128 (12)	−0.0272 (15)	0.0069 (13)
C57	0.100 (3)	0.0370 (18)	0.061 (2)	−0.033 (2)	−0.031 (2)	0.0006 (16)
C58	0.105 (3)	0.0378 (19)	0.085 (3)	−0.031 (2)	−0.045 (3)	−0.0033 (19)
C59	0.0461 (17)	0.0231 (13)	0.0394 (16)	−0.0110 (12)	−0.0090 (13)	0.0015 (11)
C60	0.0565 (19)	0.0214 (13)	0.0451 (18)	−0.0064 (13)	−0.0167 (15)	−0.0016 (12)
C61	0.0498 (17)	0.0236 (13)	0.0460 (17)	−0.0144 (13)	−0.0285 (14)	0.0059 (12)
C62	0.0390 (16)	0.0290 (15)	0.062 (2)	−0.0155 (13)	−0.0171 (15)	0.0037 (13)
C63	0.0368 (16)	0.0258 (14)	0.0568 (19)	−0.0117 (12)	−0.0138 (14)	−0.0023 (12)
C64	0.125 (4)	0.0310 (18)	0.076 (3)	−0.024 (2)	−0.064 (3)	−0.0006 (17)
C65	0.111 (3)	0.0334 (17)	0.056 (2)	−0.029 (2)	−0.043 (2)	0.0029 (15)
C66	0.0561 (19)	0.0223 (14)	0.066 (2)	−0.0139 (13)	−0.0405 (17)	0.0077 (13)
C67	0.0432 (19)	0.0324 (17)	0.110 (3)	−0.0163 (15)	−0.026 (2)	0.0129 (18)
C68	0.054 (2)	0.0326 (19)	0.137 (4)	−0.0223 (17)	−0.041 (2)	0.023 (2)
O1W	0.0384 (11)	0.0197 (9)	0.0562 (13)	−0.0066 (8)	−0.0254 (10)	0.0007 (9)
O1	0.0571 (14)	0.0420 (12)	0.0500 (13)	−0.0083 (11)	−0.0180 (11)	−0.0107 (10)
O2W	0.0375 (11)	0.0179 (9)	0.0633 (14)	−0.0048 (8)	−0.0257 (10)	−0.0008 (9)
O2	0.0478 (12)	0.0455 (12)	0.0401 (12)	−0.0209 (10)	−0.0154 (10)	0.0009 (9)
O3	0.0513 (15)	0.0436 (13)	0.097 (2)	−0.0222 (12)	−0.0063 (14)	−0.0164 (13)
O3W	0.0616 (17)	0.0551 (15)	0.0695 (17)	−0.0239 (13)	−0.0238 (14)	0.0154 (12)
O4	0.0340 (11)	0.0228 (10)	0.0764 (15)	−0.0097 (8)	−0.0205 (10)	0.0101 (9)
O5	0.0328 (10)	0.0219 (9)	0.0636 (13)	−0.0068 (8)	−0.0243 (10)	−0.0044 (8)
O6	0.098 (2)	0.0268 (11)	0.0549 (14)	−0.0243 (12)	−0.0125 (13)	−0.0026 (10)
O7	0.0610 (14)	0.0500 (13)	0.0398 (12)	−0.0395 (11)	−0.0151 (10)	0.0040 (9)
O8	0.0324 (10)	0.0223 (9)	0.0391 (11)	−0.0116 (8)	−0.0094 (8)	0.0037 (7)
O9	0.0600 (15)	0.0348 (12)	0.0775 (17)	−0.0145 (11)	−0.0355 (13)	0.0054 (11)
O10	0.0331 (10)	0.0217 (9)	0.0342 (10)	−0.0115 (8)	−0.0101 (8)	0.0062 (7)
O11	0.0507 (12)	0.0323 (10)	0.0390 (11)	−0.0254 (9)	−0.0175 (9)	0.0063 (8)
O12	0.0526 (13)	0.0313 (11)	0.0533 (13)	−0.0184 (10)	−0.0128 (10)	0.0112 (9)
O13	0.0279 (10)	0.0220 (9)	0.0616 (13)	−0.0067 (8)	−0.0152 (9)	−0.0042 (8)
O14	0.0329 (10)	0.0187 (9)	0.0632 (14)	−0.0051 (8)	−0.0085 (10)	0.0011 (8)
O15	0.0805 (17)	0.0238 (10)	0.0616 (15)	−0.0150 (11)	−0.0258 (13)	−0.0016 (10)
O16	0.0413 (12)	0.0358 (11)	0.0430 (12)	−0.0147 (9)	−0.0128 (9)	−0.0028 (9)
O17	0.0506 (13)	0.0418 (12)	0.0420 (12)	0.0038 (10)	−0.0190 (10)	−0.0069 (9)
O18	0.0747 (18)	0.0339 (13)	0.108 (2)	−0.0175 (12)	−0.0288 (16)	−0.0068 (13)

### Geometric parameters (Å, °)

**Table:**

Tm1—O1W	2.3429 (18)	C28—O12	1.379 (3)
Tm1—O5	2.3588 (17)	C28—C29	1.384 (4)
Tm1—O10	2.3621 (16)	C29—C30	1.383 (4)
Tm1—O4	2.3648 (19)	C29—H29A	0.9300
Tm1—O2	2.372 (2)	C30—H30A	0.9300
Tm1—O7	2.3866 (19)	C31—C32	1.500 (4)
Tm1—N1	2.507 (2)	C31—H31A	0.9700
Tm1—O8	2.5406 (18)	C31—H31B	0.9700
Tm1—O1	2.528 (2)	C32—O11	1.252 (3)
Tm1—C16	2.713 (3)	C32—O10	1.274 (3)
Tm1—C8	2.825 (3)	C33—C38	1.379 (4)
Tm1—C24	2.836 (3)	C33—C34	1.389 (4)
Tm2—O8	2.3052 (16)	C33—C39	1.503 (3)
Tm2—O2W	2.3454 (18)	C34—C35	1.371 (4)
Tm2—O14	2.3470 (19)	C34—H34A	0.9300
Tm2—O13	2.3719 (17)	C35—C36	1.383 (4)
Tm2—O16	2.3875 (19)	C35—H35A	0.9300
Tm2—O17	2.474 (2)	C36—O15	1.363 (3)
Tm2—O10	2.4947 (17)	C36—C37	1.379 (4)
Tm2—O11	2.5235 (18)	C37—C38	1.385 (4)
Tm2—N3	2.525 (2)	C37—H37A	0.9300
Tm2—C40	2.738 (3)	C38—H38A	0.9300
Tm2—C48	2.810 (3)	C39—C40	1.502 (4)
Tm2—C32	2.889 (3)	C39—H39A	0.9700
N1—C49	1.335 (3)	C39—H39B	0.9700
N1—C53	1.341 (3)	C40—O14	1.257 (3)
N2—C58	1.313 (5)	C40—O13	1.266 (3)
N2—C54	1.314 (5)	C41—C46	1.389 (4)
N3—C59	1.338 (3)	C41—C42	1.390 (4)
N3—C63	1.337 (3)	C41—C47	1.513 (4)
N4—C64	1.326 (5)	C42—C43	1.377 (4)
N4—C68	1.331 (5)	C42—H42A	0.9300
C1—C2	1.381 (4)	C43—C44	1.378 (4)
C1—C6	1.389 (4)	C43—H43A	0.9300
C1—C7	1.514 (4)	C44—O18	1.366 (4)
C2—C3	1.387 (4)	C44—C45	1.379 (5)
C2—H2A	0.9300	C45—C46	1.376 (4)
C3—C4	1.367 (4)	C45—H45A	0.9300
C3—H3A	0.9300	C46—H46A	0.9300
C4—C5	1.371 (4)	C47—C48	1.506 (4)
C4—O3	1.380 (3)	C47—H47A	0.9700
C5—C6	1.376 (4)	C47—H47B	0.9700
C5—H5A	0.9300	C48—O16	1.258 (3)
C6—H6A	0.9300	C48—O17	1.258 (3)
C7—C8	1.506 (4)	C49—C50	1.376 (3)
C7—H7A	0.9700	C49—H49A	0.9300
C7—H7B	0.9700	C50—C51	1.382 (4)
C8—O1	1.249 (4)	C50—H50A	0.9300
C8—O2	1.261 (3)	C51—C52	1.385 (4)
C9—C10	1.374 (4)	C51—C56	1.492 (3)
C9—C14	1.383 (4)	C52—C53	1.378 (4)
C9—C15	1.504 (4)	C52—H52A	0.9300
C10—C11	1.384 (4)	C53—H53A	0.9300
C10—H10A	0.9300	C54—C55	1.394 (4)
C11—C12	1.375 (4)	C54—H54A	0.9300
C11—H11A	0.9300	C55—C56	1.378 (4)
C12—C13	1.367 (4)	C55—H55A	0.9300
C12—O6	1.373 (3)	C56—C57	1.377 (4)
C13—C14	1.369 (4)	C57—C58	1.379 (4)
C13—H13A	0.9300	C57—H57A	0.9300
C14—H14A	0.9300	C58—H58A	0.9300
C15—C16	1.508 (4)	C59—C60	1.383 (4)
C15—H15A	0.9700	C59—H59A	0.9300
C15—H15B	0.9700	C60—C61	1.382 (4)
C16—O4	1.247 (3)	C60—H60A	0.9300
C16—O5	1.261 (3)	C61—C62	1.382 (4)
C17—C18	1.378 (5)	C61—C66	1.495 (3)
C17—C22	1.387 (4)	C62—C63	1.385 (4)
C17—C23	1.509 (4)	C62—H62A	0.9300
C18—C19	1.388 (4)	C63—H63A	0.9300
C18—H18A	0.9300	C64—C65	1.391 (4)
C19—C20	1.383 (4)	C64—H64A	0.9300
C19—H19A	0.9300	C65—C66	1.382 (5)
C20—O9	1.365 (3)	C65—H65A	0.9300
C20—C21	1.384 (4)	C66—C67	1.379 (5)
C21—C22	1.381 (4)	C67—C68	1.384 (4)
C21—H21A	0.9300	C67—H67A	0.9300
C22—H22A	0.9300	C68—H68A	0.9300
C23—C24	1.501 (4)	O1W—H1WA	0.84 (5)
C23—H23A	0.9700	O1W—H1WB	0.830 (18)
C23—H23B	0.9700	O2W—H2WA	0.83 (4)
C24—O7	1.243 (3)	O2W—H2WB	0.824 (18)
C24—O8	1.271 (3)	O3—H3B	0.8200
C25—C30	1.375 (4)	O3W—H3WB	0.84 (4)
C25—C26	1.384 (4)	O3W—H3WA	0.85 (4)
C25—C31	1.510 (4)	O6—H6B	0.8200
C26—C27	1.386 (4)	O9—H9A	0.8200
C26—H26A	0.9300	O12—H12A	0.8200
C27—C28	1.370 (4)	O15—H15C	0.8200
C27—H27A	0.9300	O18—H18B	0.8200
			
O1W—Tm1—O5	145.81 (6)	C19—C18—H18A	119.3
O1W—Tm1—O10	79.72 (6)	C20—C19—C18	119.9 (3)
O5—Tm1—O10	73.62 (6)	C20—C19—H19A	120.1
O1W—Tm1—O4	149.21 (7)	C18—C19—H19A	120.1
O5—Tm1—O4	54.84 (6)	O9—C20—C21	118.0 (3)
O10—Tm1—O4	128.08 (6)	O9—C20—C19	122.7 (3)
O1W—Tm1—O2	74.82 (7)	C21—C20—C19	119.2 (3)
O5—Tm1—O2	121.79 (7)	C20—C21—C22	120.3 (3)
O10—Tm1—O2	83.43 (6)	C20—C21—H21A	119.9
O4—Tm1—O2	116.76 (7)	C22—C21—H21A	119.9
O1W—Tm1—O7	78.28 (8)	C21—C22—C17	121.0 (3)
O5—Tm1—O7	95.13 (7)	C21—C22—H22A	119.5
O10—Tm1—O7	117.18 (6)	C17—C22—H22A	119.5
O4—Tm1—O7	76.69 (7)	C24—C23—C17	112.5 (2)
O2—Tm1—O7	142.29 (7)	C24—C23—H23A	109.1
O1W—Tm1—N1	80.66 (7)	C17—C23—H23A	109.1
O5—Tm1—N1	130.63 (7)	C24—C23—H23B	109.1
O10—Tm1—N1	153.56 (7)	C17—C23—H23B	109.1
O4—Tm1—N1	75.96 (7)	H23A—C23—H23B	107.8
O2—Tm1—N1	74.43 (7)	O7—C24—O8	119.7 (2)
O7—Tm1—N1	75.54 (7)	O7—C24—C23	120.2 (2)
O1W—Tm1—O8	73.93 (6)	O8—C24—C23	120.2 (2)
O5—Tm1—O8	75.66 (6)	O7—C24—Tm1	56.50 (14)
O10—Tm1—O8	65.26 (6)	O8—C24—Tm1	63.62 (14)
O4—Tm1—O8	103.72 (7)	C23—C24—Tm1	171.5 (2)
O2—Tm1—O8	138.88 (6)	C30—C25—C26	118.3 (2)
O7—Tm1—O8	52.23 (6)	C30—C25—C31	119.7 (3)
N1—Tm1—O8	125.07 (6)	C26—C25—C31	122.1 (3)
O1W—Tm1—O1	127.14 (7)	C25—C26—C27	120.9 (3)
O5—Tm1—O1	75.11 (7)	C25—C26—H26A	119.5
O10—Tm1—O1	91.31 (7)	C27—C26—H26A	119.5
O4—Tm1—O1	71.38 (7)	C28—C27—C26	119.8 (3)
O2—Tm1—O1	52.36 (7)	C28—C27—H27A	120.1
O7—Tm1—O1	146.35 (7)	C26—C27—H27A	120.1
N1—Tm1—O1	86.54 (7)	C27—C28—O12	122.2 (3)
O8—Tm1—O1	146.70 (6)	C27—C28—C29	120.2 (3)
O1W—Tm1—C16	164.15 (8)	O12—C28—C29	117.5 (3)
O5—Tm1—C16	27.68 (7)	C30—C29—C28	119.2 (3)
O10—Tm1—C16	100.75 (7)	C30—C29—H29A	120.4
O4—Tm1—C16	27.35 (7)	C28—C29—H29A	120.4
O2—Tm1—C16	121.03 (8)	C25—C30—C29	121.6 (3)
O7—Tm1—C16	87.61 (8)	C25—C30—H30A	119.2
N1—Tm1—C16	102.96 (7)	C29—C30—H30A	119.2
O8—Tm1—C16	91.76 (7)	C32—C31—C25	114.9 (2)
O1—Tm1—C16	68.69 (8)	C32—C31—H31A	108.5
O1W—Tm1—C8	101.08 (8)	C25—C31—H31A	108.5
O5—Tm1—C8	99.60 (8)	C32—C31—H31B	108.5
O10—Tm1—C8	89.28 (7)	C25—C31—H31B	108.5
O4—Tm1—C8	93.09 (8)	H31A—C31—H31B	107.5
O2—Tm1—C8	26.29 (8)	O11—C32—O10	119.0 (2)
O7—Tm1—C8	152.56 (7)	O11—C32—C31	122.7 (2)
N1—Tm1—C8	77.29 (7)	O10—C32—C31	118.3 (2)
O8—Tm1—C8	154.49 (6)	O11—C32—Tm2	60.65 (13)
O1—Tm1—C8	26.25 (8)	O10—C32—Tm2	59.41 (13)
C16—Tm1—C8	94.77 (9)	C31—C32—Tm2	169.0 (2)
O1W—Tm1—C24	72.60 (8)	C38—C33—C34	117.5 (2)
O5—Tm1—C24	86.92 (7)	C38—C33—C39	121.6 (3)
O10—Tm1—C24	91.46 (7)	C34—C33—C39	120.9 (2)
O4—Tm1—C24	91.64 (8)	C35—C34—C33	121.5 (3)
O2—Tm1—C24	147.41 (8)	C35—C34—H34A	119.2
O7—Tm1—C24	25.74 (6)	C33—C34—H34A	119.2
N1—Tm1—C24	99.40 (7)	C34—C35—C36	120.3 (3)
O8—Tm1—C24	26.63 (6)	C34—C35—H35A	119.8
O1—Tm1—C24	160.22 (8)	C36—C35—H35A	119.8
C16—Tm1—C24	91.56 (8)	O15—C36—C37	123.4 (3)
C8—Tm1—C24	173.38 (8)	O15—C36—C35	117.6 (3)
O8—Tm2—O2W	78.47 (6)	C37—C36—C35	119.1 (3)
O8—Tm2—O14	129.34 (6)	C36—C37—C38	120.0 (3)
O2W—Tm2—O14	143.01 (7)	C36—C37—H37A	120.0
O8—Tm2—O13	75.23 (6)	C38—C37—H37A	120.0
O2W—Tm2—O13	148.01 (6)	C33—C38—C37	121.6 (3)
O14—Tm2—O13	54.77 (6)	C33—C38—H38A	119.2
O8—Tm2—O16	79.84 (6)	C37—C38—H38A	119.2
O2W—Tm2—O16	75.44 (7)	C40—C39—C33	115.6 (2)
O14—Tm2—O16	127.30 (7)	C40—C39—H39A	108.4
O13—Tm2—O16	116.74 (7)	C33—C39—H39A	108.4
O8—Tm2—O17	99.41 (7)	C40—C39—H39B	108.4
O2W—Tm2—O17	127.36 (7)	C33—C39—H39B	108.4
O14—Tm2—O17	77.18 (7)	H39A—C39—H39B	107.4
O13—Tm2—O17	75.29 (7)	O14—C40—O13	118.7 (2)
O16—Tm2—O17	52.89 (7)	O14—C40—C39	119.2 (2)
O8—Tm2—O10	66.82 (6)	O13—C40—C39	122.1 (2)
O2W—Tm2—O10	77.23 (6)	O14—C40—Tm2	58.79 (13)
O14—Tm2—O10	91.41 (6)	O13—C40—Tm2	59.94 (13)
O13—Tm2—O10	75.88 (6)	C39—C40—Tm2	176.85 (18)
O16—Tm2—O10	140.30 (6)	C46—C41—C42	117.3 (3)
O17—Tm2—O10	150.44 (6)	C46—C41—C47	120.7 (3)
O8—Tm2—O11	115.68 (6)	C42—C41—C47	122.0 (3)
O2W—Tm2—O11	72.88 (7)	C43—C42—C41	121.6 (3)
O14—Tm2—O11	72.62 (7)	C43—C42—H42A	119.2
O13—Tm2—O11	102.67 (7)	C41—C42—H42A	119.2
O16—Tm2—O11	140.41 (6)	C44—C43—C42	120.0 (3)
O17—Tm2—O11	143.35 (7)	C44—C43—H43A	120.0
O10—Tm2—O11	51.40 (5)	C42—C43—H43A	120.0
O8—Tm2—N3	153.26 (7)	O18—C44—C43	117.8 (3)
O2W—Tm2—N3	82.95 (7)	O18—C44—C45	122.6 (3)
O14—Tm2—N3	76.24 (7)	C43—C44—C45	119.5 (3)
O13—Tm2—N3	127.58 (7)	C44—C45—C46	120.1 (3)
O16—Tm2—N3	76.97 (7)	C44—C45—H45A	119.9
O17—Tm2—N3	76.90 (7)	C46—C45—H45A	119.9
O10—Tm2—N3	127.23 (6)	C45—C46—C41	121.5 (3)
O11—Tm2—N3	76.15 (6)	C45—C46—H46A	119.2
O8—Tm2—C40	102.52 (7)	C41—C46—H46A	119.2
O2W—Tm2—C40	158.42 (8)	C48—C47—C41	112.1 (2)
O14—Tm2—C40	27.26 (6)	C48—C47—H47A	109.2
O13—Tm2—C40	27.52 (6)	C41—C47—H47A	109.2
O16—Tm2—C40	126.11 (7)	C48—C47—H47B	109.2
O17—Tm2—C40	74.05 (7)	C41—C47—H47B	109.2
O10—Tm2—C40	83.34 (7)	H47A—C47—H47B	107.9
O11—Tm2—C40	87.71 (7)	O16—C48—O17	118.9 (3)
N3—Tm2—C40	101.83 (7)	O16—C48—C47	119.8 (3)
O8—Tm2—C48	91.32 (7)	O17—C48—C47	121.3 (3)
O2W—Tm2—C48	101.03 (8)	O16—C48—Tm2	57.69 (14)
O14—Tm2—C48	101.85 (8)	O17—C48—Tm2	61.65 (15)
O13—Tm2—C48	97.49 (8)	C47—C48—Tm2	172.08 (18)
O16—Tm2—C48	26.45 (8)	N1—C49—C50	123.5 (3)
O17—Tm2—C48	26.57 (8)	N1—C49—H49A	118.3
O10—Tm2—C48	158.07 (6)	C50—C49—H49A	118.3
O11—Tm2—C48	149.54 (6)	C49—C50—C51	119.9 (3)
N3—Tm2—C48	73.48 (7)	C49—C50—H50A	120.1
C40—Tm2—C48	100.49 (8)	C51—C50—H50A	120.1
O8—Tm2—C32	90.63 (6)	C50—C51—C52	117.0 (2)
O2W—Tm2—C32	70.55 (8)	C50—C51—C56	121.3 (3)
O14—Tm2—C32	83.79 (7)	C52—C51—C56	121.7 (3)
O13—Tm2—C32	91.73 (7)	C53—C52—C51	119.7 (3)
O16—Tm2—C32	145.88 (7)	C53—C52—H52A	120.1
O17—Tm2—C32	160.80 (7)	C51—C52—H52A	120.1
O10—Tm2—C32	26.07 (6)	N1—C53—C52	123.3 (3)
O11—Tm2—C32	25.62 (6)	N1—C53—H53A	118.4
N3—Tm2—C32	101.17 (7)	C52—C53—H53A	118.4
C40—Tm2—C32	87.87 (7)	N2—C54—C55	125.0 (4)
C48—Tm2—C32	170.78 (8)	N2—C54—H54A	117.5
C49—N1—C53	116.6 (2)	C55—C54—H54A	117.5
C49—N1—Tm1	124.20 (17)	C56—C55—C54	118.3 (4)
C53—N1—Tm1	118.95 (17)	C56—C55—H55A	120.9
C58—N2—C54	115.5 (3)	C54—C55—H55A	120.9
C59—N3—C63	116.5 (2)	C57—C56—C55	117.1 (3)
C59—N3—Tm2	121.19 (17)	C57—C56—C51	122.0 (3)
C63—N3—Tm2	122.16 (18)	C55—C56—C51	120.8 (3)
C64—N4—C68	116.6 (3)	C58—C57—C56	119.3 (4)
C2—C1—C6	117.5 (3)	C58—C57—H57A	120.4
C2—C1—C7	122.4 (3)	C56—C57—H57A	120.4
C6—C1—C7	120.1 (3)	N2—C58—C57	124.7 (4)
C1—C2—C3	121.3 (3)	N2—C58—H58A	117.6
C1—C2—H2A	119.4	C57—C58—H58A	117.6
C3—C2—H2A	119.4	N3—C59—C60	123.2 (3)
C4—C3—C2	120.0 (3)	N3—C59—H59A	118.4
C4—C3—H3A	120.0	C60—C59—H59A	118.4
C2—C3—H3A	120.0	C61—C60—C59	120.4 (3)
C3—C4—C5	119.8 (3)	C61—C60—H60A	119.8
C3—C4—O3	118.5 (3)	C59—C60—H60A	119.8
C5—C4—O3	121.7 (3)	C62—C61—C60	116.4 (2)
C4—C5—C6	120.2 (3)	C62—C61—C66	122.6 (3)
C4—C5—H5A	119.9	C60—C61—C66	120.9 (3)
C6—C5—H5A	119.9	C61—C62—C63	120.0 (3)
C5—C6—C1	121.3 (3)	C61—C62—H62A	120.0
C5—C6—H6A	119.4	C63—C62—H62A	120.0
C1—C6—H6A	119.4	N3—C63—C62	123.4 (3)
C8—C7—C1	111.4 (2)	N3—C63—H63A	118.3
C8—C7—H7A	109.3	C62—C63—H63A	118.3
C1—C7—H7A	109.3	N4—C64—C65	123.4 (4)
C8—C7—H7B	109.3	N4—C64—H64A	118.3
C1—C7—H7B	109.3	C65—C64—H64A	118.3
H7A—C7—H7B	108.0	C66—C65—C64	119.4 (4)
O1—C8—O2	119.3 (3)	C66—C65—H65A	120.3
O1—C8—C7	121.3 (3)	C64—C65—H65A	120.3
O2—C8—C7	119.4 (3)	C67—C66—C65	117.3 (3)
O1—C8—Tm1	63.51 (16)	C67—C66—C61	120.9 (3)
O2—C8—Tm1	56.39 (15)	C65—C66—C61	121.7 (3)
C7—C8—Tm1	169.35 (19)	C66—C67—C68	119.1 (4)
C10—C9—C14	117.1 (3)	C66—C67—H67A	120.4
C10—C9—C15	121.9 (3)	C68—C67—H67A	120.4
C14—C9—C15	121.0 (3)	N4—C68—C67	124.0 (4)
C9—C10—C11	122.1 (3)	N4—C68—H68A	118.0
C9—C10—H10A	119.0	C67—C68—H68A	118.0
C11—C10—H10A	119.0	Tm1—O1W—H1WA	122 (3)
C12—C11—C10	119.2 (3)	Tm1—O1W—H1WB	133 (3)
C12—C11—H11A	120.4	H1WA—O1W—H1WB	104 (3)
C10—C11—H11A	120.4	C8—O1—Tm1	90.25 (18)
C13—C12—O6	117.8 (3)	Tm2—O2W—H2WA	125 (3)
C13—C12—C11	119.7 (3)	Tm2—O2W—H2WB	131 (3)
O6—C12—C11	122.5 (3)	H2WA—O2W—H2WB	104 (3)
C12—C13—C14	120.3 (3)	C8—O2—Tm1	97.32 (18)
C12—C13—H13A	119.9	C4—O3—H3B	109.5
C14—C13—H13A	119.9	H3WB—O3W—H3WA	102 (2)
C13—C14—C9	121.6 (3)	C16—O4—Tm1	92.07 (16)
C13—C14—H14A	119.2	C16—O5—Tm1	91.99 (15)
C9—C14—H14A	119.2	C12—O6—H6B	109.5
C9—C15—C16	115.4 (2)	C24—O7—Tm1	97.75 (16)
C9—C15—H15A	108.4	C24—O8—Tm2	153.90 (17)
C16—C15—H15A	108.4	C24—O8—Tm1	89.75 (15)
C9—C15—H15B	108.4	Tm2—O8—Tm1	114.13 (7)
C16—C15—H15B	108.4	C20—O9—H9A	109.5
H15A—C15—H15B	107.5	C32—O10—Tm1	142.22 (17)
O4—C16—O5	120.3 (2)	C32—O10—Tm2	94.52 (15)
O4—C16—C15	119.3 (2)	Tm1—O10—Tm2	113.79 (7)
O5—C16—C15	120.4 (2)	C32—O11—Tm2	93.73 (15)
O4—C16—Tm1	60.58 (14)	C28—O12—H12A	109.5
O5—C16—Tm1	60.33 (13)	C40—O13—Tm2	92.53 (14)
C15—C16—Tm1	170.5 (2)	C40—O14—Tm2	93.96 (15)
C18—C17—C22	118.3 (3)	C36—O15—H15C	109.5
C18—C17—C23	120.8 (3)	C48—O16—Tm2	95.86 (17)
C22—C17—C23	120.9 (3)	C48—O17—Tm2	91.78 (17)
C17—C18—C19	121.3 (3)	C44—O18—H18B	109.5
C17—C18—H18A	119.3		
			
O1W—Tm1—N1—C49	51.7 (2)	C40—Tm2—C48—O17	−5.64 (17)
O5—Tm1—N1—C49	−143.8 (2)	C53—N1—C49—C50	1.5 (4)
O10—Tm1—N1—C49	9.2 (3)	Tm1—N1—C49—C50	−173.0 (2)
O4—Tm1—N1—C49	−148.5 (2)	N1—C49—C50—C51	0.2 (4)
O2—Tm1—N1—C49	−25.0 (2)	C49—C50—C51—C52	−2.0 (4)
O7—Tm1—N1—C49	131.9 (2)	C49—C50—C51—C56	176.2 (3)
O8—Tm1—N1—C49	114.5 (2)	C50—C51—C52—C53	2.1 (4)
O1—Tm1—N1—C49	−76.8 (2)	C56—C51—C52—C53	−176.0 (3)
C16—Tm1—N1—C49	−144.0 (2)	C49—N1—C53—C52	−1.4 (4)
C8—Tm1—N1—C49	−52.0 (2)	Tm1—N1—C53—C52	173.5 (2)
C24—Tm1—N1—C49	122.2 (2)	C51—C52—C53—N1	−0.5 (5)
O1W—Tm1—N1—C53	−122.7 (2)	C58—N2—C54—C55	2.3 (6)
O5—Tm1—N1—C53	41.8 (2)	N2—C54—C55—C56	−1.2 (6)
O10—Tm1—N1—C53	−165.26 (18)	C54—C55—C56—C57	−1.2 (5)
O4—Tm1—N1—C53	37.1 (2)	C54—C55—C56—C51	175.7 (3)
O2—Tm1—N1—C53	160.6 (2)	C50—C51—C56—C57	26.3 (4)
O7—Tm1—N1—C53	−42.5 (2)	C52—C51—C56—C57	−155.6 (3)
O8—Tm1—N1—C53	−60.0 (2)	C50—C51—C56—C55	−150.5 (3)
O1—Tm1—N1—C53	108.8 (2)	C52—C51—C56—C55	27.6 (4)
C16—Tm1—N1—C53	41.5 (2)	C55—C56—C57—C58	2.3 (5)
C8—Tm1—N1—C53	133.6 (2)	C51—C56—C57—C58	−174.6 (3)
C24—Tm1—N1—C53	−52.3 (2)	C54—N2—C58—C57	−1.1 (6)
O8—Tm2—N3—C59	0.1 (3)	C56—C57—C58—N2	−1.2 (6)
O2W—Tm2—N3—C59	−46.1 (2)	C63—N3—C59—C60	−2.1 (4)
O14—Tm2—N3—C59	164.7 (2)	Tm2—N3—C59—C60	173.1 (2)
O13—Tm2—N3—C59	144.43 (19)	N3—C59—C60—C61	−1.6 (5)
O16—Tm2—N3—C59	30.6 (2)	C59—C60—C61—C62	3.9 (4)
O17—Tm2—N3—C59	85.0 (2)	C59—C60—C61—C66	−173.2 (3)
O10—Tm2—N3—C59	−114.1 (2)	C60—C61—C62—C63	−2.6 (4)
O11—Tm2—N3—C59	−120.1 (2)	C66—C61—C62—C63	174.4 (3)
C40—Tm2—N3—C59	155.3 (2)	C59—N3—C63—C62	3.4 (4)
C48—Tm2—N3—C59	57.7 (2)	Tm2—N3—C63—C62	−171.7 (2)
C32—Tm2—N3—C59	−114.6 (2)	C61—C62—C63—N3	−1.1 (5)
O8—Tm2—N3—C63	174.94 (19)	C68—N4—C64—C65	0.7 (5)
O2W—Tm2—N3—C63	128.8 (2)	N4—C64—C65—C66	1.4 (6)
O14—Tm2—N3—C63	−20.4 (2)	C64—C65—C66—C67	−2.3 (5)
O13—Tm2—N3—C63	−40.7 (2)	C64—C65—C66—C61	174.4 (3)
O16—Tm2—N3—C63	−154.6 (2)	C62—C61—C66—C67	−17.1 (4)
O17—Tm2—N3—C63	−100.2 (2)	C60—C61—C66—C67	159.7 (3)
O10—Tm2—N3—C63	60.7 (2)	C62—C61—C66—C65	166.3 (3)
O11—Tm2—N3—C63	54.8 (2)	C60—C61—C66—C65	−16.8 (4)
C40—Tm2—N3—C63	−29.8 (2)	C65—C66—C67—C68	1.3 (5)
C48—Tm2—N3—C63	−127.4 (2)	C61—C66—C67—C68	−175.4 (3)
C32—Tm2—N3—C63	60.3 (2)	C64—N4—C68—C67	−1.9 (6)
C6—C1—C2—C3	−1.3 (5)	C66—C67—C68—N4	0.9 (6)
C7—C1—C2—C3	−179.4 (3)	O2—C8—O1—Tm1	8.6 (2)
C1—C2—C3—C4	0.9 (5)	C7—C8—O1—Tm1	−169.1 (2)
C2—C3—C4—C5	0.4 (5)	O1W—Tm1—O1—C8	−7.62 (19)
C2—C3—C4—O3	−179.5 (3)	O5—Tm1—O1—C8	−158.36 (17)
C3—C4—C5—C6	−1.3 (5)	O10—Tm1—O1—C8	−85.70 (16)
O3—C4—C5—C6	178.6 (3)	O4—Tm1—O1—C8	144.27 (17)
C4—C5—C6—C1	0.9 (5)	O2—Tm1—O1—C8	−5.04 (15)
C2—C1—C6—C5	0.4 (5)	O7—Tm1—O1—C8	125.08 (17)
C7—C1—C6—C5	178.6 (3)	N1—Tm1—O1—C8	67.92 (16)
C2—C1—C7—C8	−47.3 (4)	O8—Tm1—O1—C8	−129.01 (16)
C6—C1—C7—C8	134.6 (3)	C16—Tm1—O1—C8	173.27 (18)
C1—C7—C8—O1	96.2 (3)	C24—Tm1—O1—C8	176.31 (19)
C1—C7—C8—O2	−81.5 (3)	O1—C8—O2—Tm1	−9.3 (3)
C1—C7—C8—Tm1	−17.7 (13)	C7—C8—O2—Tm1	168.5 (2)
O1W—Tm1—C8—O1	173.82 (15)	O1W—Tm1—O2—C8	−177.10 (16)
O5—Tm1—C8—O1	21.19 (16)	O5—Tm1—O2—C8	35.73 (17)
O10—Tm1—C8—O1	94.44 (16)	O10—Tm1—O2—C8	101.80 (16)
O4—Tm1—C8—O1	−33.66 (16)	O4—Tm1—O2—C8	−27.78 (17)
O2—Tm1—C8—O1	171.0 (3)	O7—Tm1—O2—C8	−131.12 (16)
O7—Tm1—C8—O1	−100.3 (2)	N1—Tm1—O2—C8	−92.78 (16)
N1—Tm1—C8—O1	−108.52 (17)	O8—Tm1—O2—C8	141.22 (14)
O8—Tm1—C8—O1	98.0 (2)	O1—Tm1—O2—C8	5.03 (15)
C16—Tm1—C8—O1	−6.29 (17)	C16—Tm1—O2—C8	3.19 (18)
O1W—Tm1—C8—O2	2.85 (16)	C24—Tm1—O2—C8	−175.81 (15)
O5—Tm1—C8—O2	−149.78 (15)	O5—C16—O4—Tm1	9.1 (3)
O10—Tm1—C8—O2	−76.53 (15)	C15—C16—O4—Tm1	−169.1 (3)
O4—Tm1—C8—O2	155.37 (15)	O1W—Tm1—O4—C16	−147.77 (17)
O7—Tm1—C8—O2	88.7 (2)	O5—Tm1—O4—C16	−5.15 (16)
N1—Tm1—C8—O2	80.51 (16)	O10—Tm1—O4—C16	2.9 (2)
O8—Tm1—C8—O2	−73.0 (2)	O2—Tm1—O4—C16	106.36 (17)
O1—Tm1—C8—O2	−171.0 (3)	O7—Tm1—O4—C16	−111.35 (18)
C16—Tm1—C8—O2	−177.26 (15)	N1—Tm1—O4—C16	170.50 (19)
O1W—Tm1—C8—C7	−67.1 (12)	O8—Tm1—O4—C16	−66.22 (18)
O5—Tm1—C8—C7	140.3 (12)	O1—Tm1—O4—C16	79.44 (17)
O10—Tm1—C8—C7	−146.4 (12)	C8—Tm1—O4—C16	94.43 (18)
O4—Tm1—C8—C7	85.5 (12)	C24—Tm1—O4—C16	−90.22 (18)
O2—Tm1—C8—C7	−69.9 (12)	O4—C16—O5—Tm1	−9.2 (3)
O7—Tm1—C8—C7	18.8 (13)	C15—C16—O5—Tm1	169.1 (3)
N1—Tm1—C8—C7	10.6 (12)	O1W—Tm1—O5—C16	151.51 (16)
O8—Tm1—C8—C7	−142.9 (11)	O10—Tm1—O5—C16	−168.29 (17)
O1—Tm1—C8—C7	119.1 (12)	O4—Tm1—O5—C16	5.09 (16)
C16—Tm1—C8—C7	112.8 (12)	O2—Tm1—O5—C16	−97.11 (17)
C14—C9—C10—C11	1.3 (5)	O7—Tm1—O5—C16	74.86 (17)
C15—C9—C10—C11	−178.9 (3)	N1—Tm1—O5—C16	−0.5 (2)
C9—C10—C11—C12	−0.5 (5)	O8—Tm1—O5—C16	123.74 (17)
C10—C11—C12—C13	−0.6 (5)	O1—Tm1—O5—C16	−72.39 (17)
C10—C11—C12—O6	179.1 (3)	C8—Tm1—O5—C16	−81.91 (17)
O6—C12—C13—C14	−178.8 (3)	C24—Tm1—O5—C16	99.28 (17)
C11—C12—C13—C14	0.8 (5)	O8—C24—O7—Tm1	−8.2 (3)
C12—C13—C14—C9	0.0 (5)	C23—C24—O7—Tm1	170.8 (3)
C10—C9—C14—C13	−1.0 (5)	O1W—Tm1—O7—C24	−74.15 (18)
C15—C9—C14—C13	179.1 (3)	O5—Tm1—O7—C24	71.91 (18)
C10—C9—C15—C16	−96.8 (4)	O10—Tm1—O7—C24	−2.3 (2)
C14—C9—C15—C16	83.1 (4)	O4—Tm1—O7—C24	123.93 (18)
C9—C15—C16—O4	172.4 (3)	O2—Tm1—O7—C24	−119.29 (18)
C9—C15—C16—O5	−5.9 (4)	N1—Tm1—O7—C24	−157.39 (19)
O1W—Tm1—C16—O4	92.1 (3)	O8—Tm1—O7—C24	4.50 (16)
O5—Tm1—C16—O4	170.9 (3)	O1—Tm1—O7—C24	142.60 (17)
O10—Tm1—C16—O4	−177.66 (17)	C16—Tm1—O7—C24	98.57 (18)
O2—Tm1—C16—O4	−88.90 (18)	C8—Tm1—O7—C24	−165.66 (19)
O7—Tm1—C16—O4	65.12 (17)	O7—C24—O8—Tm2	164.6 (3)
N1—Tm1—C16—O4	−9.45 (18)	C23—C24—O8—Tm2	−14.4 (6)
O8—Tm1—C16—O4	117.20 (17)	Tm1—C24—O8—Tm2	157.0 (4)
O1—Tm1—C16—O4	−90.47 (18)	O7—C24—O8—Tm1	7.6 (3)
C8—Tm1—C16—O4	−87.49 (17)	C23—C24—O8—Tm1	−171.4 (3)
C24—Tm1—C16—O4	90.56 (17)	O2W—Tm2—O8—C24	124.6 (4)
O1W—Tm1—C16—O5	−78.8 (3)	O14—Tm2—O8—C24	−82.9 (4)
O10—Tm1—C16—O5	11.43 (17)	O13—Tm2—O8—C24	−73.8 (4)
O4—Tm1—C16—O5	−170.9 (3)	O16—Tm2—O8—C24	47.5 (4)
O2—Tm1—C16—O5	100.19 (16)	O17—Tm2—O8—C24	−1.8 (4)
O7—Tm1—C16—O5	−105.80 (16)	O10—Tm2—O8—C24	−154.5 (4)
N1—Tm1—C16—O5	179.63 (16)	O11—Tm2—O8—C24	−171.0 (4)
O8—Tm1—C16—O5	−53.71 (16)	N3—Tm2—O8—C24	77.7 (4)
O1—Tm1—C16—O5	98.62 (17)	C40—Tm2—O8—C24	−77.4 (4)
C8—Tm1—C16—O5	101.60 (16)	C48—Tm2—O8—C24	23.6 (4)
C24—Tm1—C16—O5	−80.35 (16)	C32—Tm2—O8—C24	−165.4 (4)
C22—C17—C18—C19	0.5 (4)	O2W—Tm2—O8—Tm1	−80.78 (8)
C23—C17—C18—C19	179.4 (3)	O14—Tm2—O8—Tm1	71.69 (11)
C17—C18—C19—C20	−0.2 (4)	O13—Tm2—O8—Tm1	80.83 (8)
C18—C19—C20—O9	−179.8 (3)	O16—Tm2—O8—Tm1	−157.84 (9)
C18—C19—C20—C21	−0.3 (4)	O17—Tm2—O8—Tm1	152.77 (7)
O9—C20—C21—C22	−179.9 (3)	O10—Tm2—O8—Tm1	0.12 (6)
C19—C20—C21—C22	0.6 (4)	O11—Tm2—O8—Tm1	−16.35 (10)
C20—C21—C22—C17	−0.3 (4)	N3—Tm2—O8—Tm1	−127.69 (13)
C18—C17—C22—C21	−0.2 (4)	C40—Tm2—O8—Tm1	77.17 (8)
C23—C17—C22—C21	−179.1 (3)	C48—Tm2—O8—Tm1	178.22 (9)
C18—C17—C23—C24	−78.8 (4)	C32—Tm2—O8—Tm1	−10.79 (8)
C22—C17—C23—C24	100.2 (4)	O1W—Tm1—O8—C24	83.11 (16)
C17—C23—C24—O7	−46.5 (4)	O5—Tm1—O8—C24	−112.70 (16)
C17—C23—C24—O8	132.5 (3)	O10—Tm1—O8—C24	169.00 (17)
O1W—Tm1—C24—O7	99.21 (18)	O4—Tm1—O8—C24	−65.09 (16)
O5—Tm1—C24—O7	−108.54 (18)	O2—Tm1—O8—C24	125.01 (16)
O10—Tm1—C24—O7	177.96 (18)	O7—Tm1—O8—C24	−4.36 (15)
O4—Tm1—C24—O7	−53.88 (18)	N1—Tm1—O8—C24	17.22 (18)
O2—Tm1—C24—O7	97.9 (2)	O1—Tm1—O8—C24	−141.97 (16)
N1—Tm1—C24—O7	22.17 (19)	C16—Tm1—O8—C24	−89.97 (16)
O8—Tm1—C24—O7	−172.1 (3)	C8—Tm1—O8—C24	165.11 (19)
O1—Tm1—C24—O7	−84.1 (3)	O1W—Tm1—O8—Tm2	−86.02 (8)
C16—Tm1—C24—O7	−81.24 (18)	O5—Tm1—O8—Tm2	78.17 (8)
O1W—Tm1—C24—O8	−88.74 (15)	O10—Tm1—O8—Tm2	−0.13 (6)
O5—Tm1—C24—O8	63.52 (15)	O4—Tm1—O8—Tm2	125.78 (8)
O10—Tm1—C24—O8	−9.99 (15)	O2—Tm1—O8—Tm2	−44.12 (12)
O4—Tm1—C24—O8	118.18 (15)	O7—Tm1—O8—Tm2	−173.49 (12)
O2—Tm1—C24—O8	−90.04 (18)	N1—Tm1—O8—Tm2	−151.91 (8)
O7—Tm1—C24—O8	172.1 (3)	O1—Tm1—O8—Tm2	48.90 (15)
N1—Tm1—C24—O8	−165.78 (14)	C16—Tm1—O8—Tm2	100.90 (8)
O1—Tm1—C24—O8	88.0 (3)	C8—Tm1—O8—Tm2	−4.0 (2)
C16—Tm1—C24—O8	90.81 (15)	C24—Tm1—O8—Tm2	−169.13 (19)
C30—C25—C26—C27	−0.9 (4)	O11—C32—O10—Tm1	−151.4 (2)
C31—C25—C26—C27	178.0 (3)	C31—C32—O10—Tm1	28.0 (4)
C25—C26—C27—C28	−0.4 (5)	Tm2—C32—O10—Tm1	−139.7 (3)
C26—C27—C28—O12	−179.0 (3)	O11—C32—O10—Tm2	−11.7 (3)
C26—C27—C28—C29	1.2 (4)	C31—C32—O10—Tm2	167.7 (2)
C27—C28—C29—C30	−0.6 (4)	O1W—Tm1—O10—C32	−147.8 (3)
O12—C28—C29—C30	179.6 (3)	O5—Tm1—O10—C32	53.9 (3)
C26—C25—C30—C29	1.5 (4)	O4—Tm1—O10—C32	47.0 (3)
C31—C25—C30—C29	−177.4 (3)	O2—Tm1—O10—C32	−72.1 (3)
C28—C29—C30—C25	−0.8 (5)	O7—Tm1—O10—C32	141.2 (3)
C30—C25—C31—C32	75.9 (4)	N1—Tm1—O10—C32	−105.1 (3)
C26—C25—C31—C32	−103.0 (3)	O8—Tm1—O10—C32	135.3 (3)
C25—C31—C32—O11	11.5 (4)	O1—Tm1—O10—C32	−20.2 (3)
C25—C31—C32—O10	−167.8 (3)	C16—Tm1—O10—C32	48.4 (3)
C25—C31—C32—Tm2	−93.1 (9)	C8—Tm1—O10—C32	−46.4 (3)
O8—Tm2—C32—O11	−168.37 (16)	C24—Tm1—O10—C32	140.2 (3)
O2W—Tm2—C32—O11	−90.83 (16)	O1W—Tm1—O10—Tm2	77.05 (8)
O14—Tm2—C32—O11	62.11 (16)	O5—Tm1—O10—Tm2	−81.32 (8)
O13—Tm2—C32—O11	116.39 (16)	O4—Tm1—O10—Tm2	−88.20 (10)
O16—Tm2—C32—O11	−95.71 (18)	O2—Tm1—O10—Tm2	152.75 (8)
O17—Tm2—C32—O11	69.7 (3)	O7—Tm1—O10—Tm2	6.03 (10)
O10—Tm2—C32—O11	168.3 (3)	N1—Tm1—O10—Tm2	119.75 (14)
N3—Tm2—C32—O11	−12.51 (17)	O8—Tm1—O10—Tm2	0.12 (6)
C40—Tm2—C32—O11	89.13 (16)	O1—Tm1—O10—Tm2	−155.38 (8)
O8—Tm2—C32—O10	23.34 (15)	C16—Tm1—O10—Tm2	−86.83 (9)
O2W—Tm2—C32—O10	100.88 (15)	C8—Tm1—O10—Tm2	178.45 (9)
O14—Tm2—C32—O10	−106.19 (15)	C24—Tm1—O10—Tm2	5.03 (8)
O13—Tm2—C32—O10	−51.90 (15)	O8—Tm2—O10—C32	−154.47 (16)
O16—Tm2—C32—O10	96.00 (17)	O2W—Tm2—O10—C32	−71.70 (15)
O17—Tm2—C32—O10	−98.6 (2)	O14—Tm2—O10—C32	72.75 (15)
O11—Tm2—C32—O10	−168.3 (3)	O13—Tm2—O10—C32	125.80 (16)
N3—Tm2—C32—O10	179.20 (14)	O16—Tm2—O10—C32	−119.15 (16)
C40—Tm2—C32—O10	−79.16 (15)	O17—Tm2—O10—C32	138.77 (17)
O8—Tm2—C32—C31	−57.4 (9)	O11—Tm2—O10—C32	6.45 (14)
O2W—Tm2—C32—C31	20.1 (9)	N3—Tm2—O10—C32	−0.98 (18)
O14—Tm2—C32—C31	173.0 (9)	C40—Tm2—O10—C32	98.83 (15)
O13—Tm2—C32—C31	−132.7 (9)	C48—Tm2—O10—C32	−159.6 (2)
O16—Tm2—C32—C31	15.2 (9)	O8—Tm2—O10—Tm1	−0.13 (6)
O17—Tm2—C32—C31	−179.4 (8)	O2W—Tm2—O10—Tm1	82.63 (8)
O10—Tm2—C32—C31	−80.8 (9)	O14—Tm2—O10—Tm1	−132.91 (8)
O11—Tm2—C32—C31	110.9 (9)	O13—Tm2—O10—Tm1	−79.87 (8)
N3—Tm2—C32—C31	98.4 (9)	O16—Tm2—O10—Tm1	35.19 (13)
C40—Tm2—C32—C31	−159.9 (9)	O17—Tm2—O10—Tm1	−66.90 (16)
C38—C33—C34—C35	−0.3 (4)	O11—Tm2—O10—Tm1	160.78 (11)
C39—C33—C34—C35	179.3 (3)	N3—Tm2—O10—Tm1	153.35 (8)
C33—C34—C35—C36	0.3 (5)	C40—Tm2—O10—Tm1	−106.83 (8)
C34—C35—C36—O15	179.9 (3)	C48—Tm2—O10—Tm1	−5.2 (2)
C34—C35—C36—C37	−0.2 (5)	C32—Tm2—O10—Tm1	154.34 (19)
O15—C36—C37—C38	180.0 (3)	O10—C32—O11—Tm2	11.5 (3)
C35—C36—C37—C38	0.0 (4)	C31—C32—O11—Tm2	−167.8 (2)
C34—C33—C38—C37	0.2 (4)	O8—Tm2—O11—C32	12.93 (18)
C39—C33—C38—C37	−179.4 (3)	O2W—Tm2—O11—C32	80.59 (16)
C36—C37—C38—C33	0.0 (4)	O14—Tm2—O11—C32	−112.98 (16)
C38—C33—C39—C40	−114.8 (3)	O13—Tm2—O11—C32	−66.60 (16)
C34—C33—C39—C40	65.6 (4)	O16—Tm2—O11—C32	118.86 (16)
C33—C39—C40—O14	172.5 (2)	O17—Tm2—O11—C32	−148.89 (15)
C33—C39—C40—O13	−8.7 (4)	O10—Tm2—O11—C32	−6.55 (14)
O8—Tm2—C40—O14	−170.72 (15)	N3—Tm2—O11—C32	167.36 (17)
O2W—Tm2—C40—O14	−80.5 (2)	C40—Tm2—O11—C32	−89.86 (16)
O13—Tm2—C40—O14	−178.4 (3)	C48—Tm2—O11—C32	163.19 (17)
O16—Tm2—C40—O14	102.81 (16)	O14—C40—O13—Tm2	1.6 (3)
O17—Tm2—C40—O14	92.92 (16)	C39—C40—O13—Tm2	−177.2 (2)
O10—Tm2—C40—O14	−106.30 (16)	O8—Tm2—O13—C40	−172.25 (16)
O11—Tm2—C40—O14	−54.91 (16)	O2W—Tm2—O13—C40	−136.56 (16)
N3—Tm2—C40—O14	20.42 (17)	O14—Tm2—O13—C40	−0.90 (15)
C48—Tm2—C40—O14	95.54 (16)	O16—Tm2—O13—C40	117.45 (15)
C32—Tm2—C40—O14	−80.55 (16)	O17—Tm2—O13—C40	83.63 (16)
O8—Tm2—C40—O13	7.68 (16)	O10—Tm2—O13—C40	−102.94 (16)
O2W—Tm2—C40—O13	97.9 (2)	O11—Tm2—O13—C40	−58.66 (16)
O14—Tm2—C40—O13	178.4 (3)	N3—Tm2—O13—C40	23.48 (18)
O16—Tm2—C40—O13	−78.79 (16)	C48—Tm2—O13—C40	98.36 (16)
O17—Tm2—C40—O13	−88.68 (16)	C32—Tm2—O13—C40	−82.05 (16)
O10—Tm2—C40—O13	72.10 (15)	O13—C40—O14—Tm2	−1.6 (3)
O11—Tm2—C40—O13	123.49 (15)	C39—C40—O14—Tm2	177.2 (2)
N3—Tm2—C40—O13	−161.18 (15)	O8—Tm2—O14—C40	11.7 (2)
C48—Tm2—C40—O13	−86.06 (16)	O2W—Tm2—O14—C40	142.93 (15)
C32—Tm2—C40—O13	97.85 (15)	O13—Tm2—O14—C40	0.91 (15)
C46—C41—C42—C43	−0.4 (4)	O16—Tm2—O14—C40	−97.99 (16)
C47—C41—C42—C43	177.4 (3)	O17—Tm2—O14—C40	−79.99 (16)
C41—C42—C43—C44	−1.5 (5)	O10—Tm2—O14—C40	72.48 (16)
C42—C43—C44—O18	−178.6 (3)	O11—Tm2—O14—C40	121.05 (17)
C42—C43—C44—C45	2.3 (5)	N3—Tm2—O14—C40	−159.41 (17)
O18—C44—C45—C46	179.7 (3)	C48—Tm2—O14—C40	−89.94 (16)
C43—C44—C45—C46	−1.3 (5)	C32—Tm2—O14—C40	97.45 (16)
C44—C45—C46—C41	−0.6 (5)	O17—C48—O16—Tm2	7.7 (3)
C42—C41—C46—C45	1.4 (4)	C47—C48—O16—Tm2	−171.2 (2)
C47—C41—C46—C45	−176.4 (3)	O8—Tm2—O16—C48	−114.38 (15)
C46—C41—C47—C48	106.9 (3)	O2W—Tm2—O16—C48	165.02 (16)
C42—C41—C47—C48	−70.8 (4)	O14—Tm2—O16—C48	17.93 (18)
C41—C47—C48—O16	59.2 (4)	O13—Tm2—O16—C48	−46.73 (16)
C41—C47—C48—O17	−119.7 (3)	O17—Tm2—O16—C48	−4.27 (14)
O8—Tm2—C48—O16	63.74 (15)	O10—Tm2—O16—C48	−147.06 (14)
O2W—Tm2—C48—O16	−14.77 (16)	O11—Tm2—O16—C48	127.32 (15)
O14—Tm2—C48—O16	−165.51 (14)	N3—Tm2—O16—C48	79.03 (15)
O13—Tm2—C48—O16	139.02 (15)	C40—Tm2—O16—C48	−16.22 (18)
O17—Tm2—C48—O16	172.4 (3)	C32—Tm2—O16—C48	169.77 (14)
O10—Tm2—C48—O16	68.4 (3)	O16—C48—O17—Tm2	−7.4 (2)
O11—Tm2—C48—O16	−89.7 (2)	C47—C48—O17—Tm2	171.5 (2)
N3—Tm2—C48—O16	−93.92 (15)	O8—Tm2—O17—C48	73.80 (16)
C40—Tm2—C48—O16	166.73 (15)	O2W—Tm2—O17—C48	−8.83 (19)
O8—Tm2—C48—O17	−108.62 (16)	O14—Tm2—O17—C48	−157.79 (17)
O2W—Tm2—C48—O17	172.86 (16)	O13—Tm2—O17—C48	145.70 (17)
O14—Tm2—C48—O17	22.12 (17)	O16—Tm2—O17—C48	4.25 (14)
O13—Tm2—C48—O17	−33.35 (16)	O10—Tm2—O17—C48	132.70 (16)
O16—Tm2—C48—O17	−172.4 (3)	O11—Tm2—O17—C48	−122.76 (16)
O10—Tm2—C48—O17	−103.9 (2)	N3—Tm2—O17—C48	−79.18 (16)
O11—Tm2—C48—O17	97.9 (2)	C40—Tm2—O17—C48	174.23 (17)
N3—Tm2—C48—O17	93.71 (17)	C32—Tm2—O17—C48	−165.55 (18)

### Hydrogen-bond geometry (Å, °)

**Table:**

*D*—H···*A*	*D*—H	H···*A*	*D*···*A*	*D*—H···*A*
O3—H3B···O12^i^	0.82	1.93	2.741 (3)	169
O6—H6B···O3W^ii^	0.82	1.86	2.646 (3)	161
O9—H9A···O17^iii^	0.82	1.86	2.677 (3)	173
O12—H12A···O11^iv^	0.82	1.94	2.752 (3)	168
O15—H15C···O6^v^	0.82	1.90	2.717 (3)	175
O18—H18B···O9^ii^	0.82	1.95	2.766 (3)	173
O2W—H2WA···O5	0.83 (4)	1.99 (2)	2.733 (3)	150 (4)
O2W—H2WB···N2^ii^	0.82 (2)	2.04 (2)	2.836 (3)	164 (4)
O3W—H3WB···O3	0.84 (4)	1.97 (4)	2.793 (3)	166 (4)
O1W—H1WA···O13	0.84 (5)	1.95 (2)	2.733 (2)	157 (4)
O1W—H1WB···N4^i^	0.83 (2)	1.96 (2)	2.780 (3)	169 (4)
O3W—H3WA···O1^vi^	0.85 (4)	1.93 (4)	2.778 (3)	174 (5)

Symmetry codes: (i) *x*, *y*+1, *z*; (ii) *x*, *y*−1, *z*; (iii) −*x*, −*y*+1, −*z*; (iv) −*x*, −*y*, −*z*+1; (v) *x*−1, *y*+1, *z*; (vi) −*x*+1, −*y*+1, −*z*+1.

## References

[bb1] Bruker (2006). *APEX2* and *SAINT* . Bruker AXS Inc., Madison, Wisconsin, USA.

[bb2] Fang, R.-Q. & Zhang, X.-M. (2006). *Inorg. Chem.* **45**, 4801–4810. 10.1021/ic052099m 16749845

[bb3] Liu, J.-L., Li, H.-Q. & Zhao, G.-L. (2010). *Acta Cryst.* E**66**, m9.

[bb4] Sheldrick, G. M. (1996). *SADABS* University of Göttingen, Germany.

[bb5] Sheldrick, G. M. (2008). *Acta Cryst.* A**64**, 112–122. 10.1107/S0108767307043930 18156677

[bb6] Wang, G.-H., Lei, Y.-Q. & Wang, N. (2010). *Cryst. Growth Des.* **10** 4060–4067.

[bb7] Wang, X.-X. & Sevov, S. (2008). *Inorg. Chem.* **47**, 1037–1043. 10.1021/ic701893z 18095675

